# Irradiation-based design of mechanically resistant microstructures tuned via multiscale phase-field modeling

**DOI:** 10.1038/s41598-018-28685-3

**Published:** 2018-07-06

**Authors:** Gilles Demange, Sylvain Dépinoy, Laurence Lunéville, David Simeone, Vassilis Pontikis

**Affiliations:** 1 GPM, UMR CNRS 6643, University of Rouen, 76575 Saint Étienne du Rouvray, France; 2 4MAT, Materials Engineering, Characterization, Processing and Recycling, Université Libre de Bruxelles, 50 Avenue FD Roosevelt, CP194/03, B-1050 Brussels, Belgium; 3 CEA/DEN/SERMA and LRC CARMEN CEA-CNRS-Centrale supelec/SPMS, Université Paris-Saclay, 91191 Gif-sur-Yvette, France; 4 CEA/DEN/SRMA and LRC CARMEN CEA-CNRS-Centrale supelec/SPMS, Université Paris-Saclay, 91191 Gif-sur-Yvette, France; 5 CEA, DEN/DMN/SRMA and DRF/IRAMIS, Université Paris-Saclay, 91191 Gif-sur-Yvette, France

## Abstract

We present a multi-scale phase field modeling of stationary microstructures produced under 1 MeV krypton ion irradiation in a phase separating concentrated solid solution of silver and copper. We show that the mixture reaches ultimately a stationary micro-structural state made of phase domains with composition and size distribution mapped to the values of the incident flux of particles and of the temperature, variables that help defining a non equilibrium phase-diagram for the irradiated alloy. The modeling predicts the formation of diverse microstructures likely connected to spinodal hardening, thus opening the perspective of the on-purpose tuning of mechanically resistant microstructures and the preparation of metastable alloys with mechanical properties improved by comparison to counterparts obtained via classical thermo-mechanical treatments.

## Introduction

Patterned microstructures forming in driven materials have been for long a hot subject of applicative research since it is foreseen that thereby new preparation routes can emerge for industrial materials with desired physical properties, surpassing these obtained via classical preparation routes^1,2^. Stationary irradiation microstructures are made of phase domains with composition and size distribution controlled by the temperature, the flux and the nominal composition of the alloy. Under irradiation at finite temperatures, these have been shown to stem from competing mass transfer processes and from the large difference existing between the characteristic length scales of the associated non local forces^3,4^. Examples of irradiation microstructures are periodic defect walls^5^, bubble or void lattices^6,7^ and periodic composition modulations in concentrated alloys^8^.

Early suggestions for micro-structural engineering targeting the preparation of mechanically resistant materials were based on the known benefit of homogenizing an initially heterogeneous microstructure via ion-beam mixing^9–12^. A challenging step further is to requesting the preparation of homogeneous microstructures with tuned composition, size distribution of phases and/or defect lattices. This is a critical issue for microelectronics, nuclear energy and other applications. Indeed, materials for microelectronics should combine electronic properties with mechanical strength^13,14^ whereas duplex stainless steels for nuclear applications should resist hardening and embrittlement due to the *δ*-ferrite phase decomposing under neutron irradiation (spinodal decomposition)^15–17^, possibly related to spinodal hardening increasing the yield stress^8,18,19^. In this context, the present study aims at providing a robust methodology for linking the external parameters of irradiation, such as the temperature, the irradiation flux and the energy of incident ions, to the resulting change in the yield stress of the irradiated alloy. To this end, the phase-field method is here adopted^20,21^, referred hereafter to as PF, for it efficiently integrates the very different space and time scales of the processes leading to patterning in irradiated materials and has been shown capable of reproducing the experimental irradiation microstructures^22^. It is worth noting that the present study has been considerably simplified by previous works from the literature, having explored this subject either, via a similar methodology or by using the Kinetic Monte-Carlo method (KMC)^23–29^. These works have shown that experimental facts, such as the formation of cherry pits under irradiation, are qualitatively reproduced^30^, without attempting any quantitative comparison of their findings with experimental observations of the size distribution and the composition of stationary phase domains. Moreover, little is known about the mechanical strength of the irradiated alloy and the variety of microstructures resulting from changes in the experimental parameters such as, the nominal composition, the temperature, the flux and the energy of incident ions, although this knowledge is the prerequisite for effectively operating microstructural engineering^31^.

Unlike others, the present work shows that at any value of the nominal composition of a binary alloy, stationary irradiation microstructures are precisely located within a pseudo-phase diagram spanned by the temperature and the irradiation flux, which knowledge allows for identifying the regions triggering spinodal hardening, while enabling the flexible tailoring of microstructural features connected with the macroscopic mechanical behavior. For the illustration of the methodology developed in the present study, the case is considered of a concentrated random solution of silver (Ag) and copper (Cu) under 1 Mev krypton (Kr) ion irradiation. This alloy possess a large miscibility gap, has been extensively studied in the literature and is well suited for X-ray spectroscopy investigations of the microstructure since the difference between Ag and Cu absorption factors facilitates identifying their distributions^32^.

In the following, the previously developed PF model of the alloy under ion irradiation is briefly presented together with the parameterization of the free-energy functional, appropriately chosen to reproduce the experimental phase diagram at equilibrium^33,34^. Under ion irradiation, the calculated pseudo-phase diagram is then presented together with the types of emerging stationary states at various temperature and ion flux values. Therefrom, the experimental conditions likely to yield the mechanical behavior needed for operating the microstructural design are selected. Moreover, the size distribution, the composition and the periodicity of precipitates are given in experimentally accessible units as functions of the temperature and the irradiation flux. Finally, an explicit connexion is established between microstructures and spinodal hardening.

## Quantitative phase-field model

### Phase-field model under irradiation

Under ion irradiation, the evolution of a binary solution is customarily described at the mesoscopic scale via the kinetic equation^33^:

1 $$\frac{\partial c}{\partial t}=M{\nabla }^{2}[\frac{\delta  {\mathcal L} (c)}{\delta c}],$$

where *c*(***r***, *t*) represents the coarse-grained concentration of species, *M*, is the atomic mobility and, ${\mathcal L} (c)$, is a non-equilibrium effective free energy defined by:

2 $$ {\mathcal L} (c)={\int }_{{\rm{\Omega }}}[{f}_{h}(c)+\frac{\kappa }{2}|\nabla c{|}^{2}+\frac{{\rm{\Gamma }}}{2M}\{g\ast c\}\,(r,t)c(r,t)]{\rm{d}}{\rm{\Omega }}\mathrm{.}$$

In this equation, ${f}_{h}(c)={a}_{2}(T)(c-{c}_{L}{)}^{2}\mathrm{/2}+{a}_{3}(T)(c-{c}_{L}{)}^{3}\mathrm{/3}+{a}_{4}(T)(c-{c}_{L}{)}^{4}\mathrm{/4}$ is the Landau polynomial expansion of the bulk free energy near the concentration, *c*_*L*_, in the liquid. Unlike the standard Cahn-Hilliard equation^35^, the presence in, *f*_*h*_, of the cubic term allows modeling of both, first and second order phase transitions^36^. However, this expression of the free-energy density neglects elastic effects relating to the large difference between effective diameters of Ag and Cu atoms. It is worth mentioning that kinetic correlations affecting the atomic mobility, such as these recently proposed in improvement of the Cahn-Hilliard equation, have not been addressed by the present work^37^.

The term $\kappa |\nabla c{|}^{2}\mathrm{/2}$, referred hereafter to as the Ginzburg term^20^, expresses the additional energy associated with emerging chemical interfaces during phase separation, with stiffness coefficient, *κ*. The last term in ${\mathcal L}$ represents the impact of compositional changes due to the relocation of species during irradiation and is therefore temperature independent^38^. The Green function, *g*, satisfies $-{\nabla }^{2}g=\delta -{p}_{R}$, where, *p*_*R*_, is the probability distribution of displacements of species occurring in sub-cascades with relocation frequency, ${\rm{\Gamma }}$^23^ and *δ*, represents the Dirac impulse. This frequency, is usually taken as position independent within the reasonable approximation that cascades homogeneously overlap and that the volume fraction of cascades covered by sub-cascades exceeds the percolation threshold^39^. Since collision cascades last a hundred of picoseconds, whereas patterns evolve slowly at the time scale of diffusion, the microstructure is sensitive only to the time-averaged ballistic effects of cascades. Accordingly, Γ = *σ*^*r*^Φ, where Φ is the irradiation flux, and *σ*^*r*^, is the effective cross-section of relocation events characterizing the efficiency of ion irradiation to inducing ballistic relocations. Under the same assumptions, the probability distribution, *p*_*R*_, is a one variable function of the relocation distance, *r*, customarily taken as a decreasing exponential with decay constant, the average relocation distance, *R*. equation 1 is easily put in adimensional form with characteristic time and length constants, ${t}_{0}=\kappa /(M{a}_{4}^{2}{\alpha }^{4}{V}_{{\rm{at}}})$, and ${l}_{0}=\sqrt{\kappa /{a}_{4}}/\alpha$, where $\alpha \simeq 0.5$ at low temperatures, and *V*_at_ is the average atomic volume in the alloy. PF simulations consisted in solving equation 1 in reduced coordinates by means of the Spectal-Eyre (SE) scheme^40^ on a 1000 × 1000 grid, with reduced space and time steps, Δ*x* = 0.5 and Δ*t* = 0.1. PF simulations have been performed in two dimensions, assuming that no determinant insight might be added by 3D simulations. However, implementing the 3D PF procedure from the existing 2D model presents no major challenge. Afterwards, the conversion of the results in real units is made with values of the above parameters representative of AgCu under 1 MeV krypton ion irradiation.

### Phase-field parametrization

In this section, the parametrization procedure of the adimensional PF model is recalled. For further details and numerics, are given in^33^.

#### Bulk free energy

PF modeling of AgCu at equilibrium, consisted in numerically determining the coefficients of the bulk free energy density, *f*_*h*_(*c*), such as to fit the experimental phase diagram of the alloy^34,41^. To this end the stationary numerical solutions of equation 1 have been determined at several concentration and temperature values along the solubility limit^33^.

#### Interface and Ginzburg term

The energetic cost of diffuse interfaces forming in the alloy during phase separation is controlled by the stiffness coefficient, *κ*, which value is not available from experiments (see equation 2). In substitution, its value has been estimated from fits of PF composition profiles across a (100) semi-coherent interface between crystalline Ag and Cu on to counterparts obtained via Grand Canonical Monte Carlo (GCMC) simulations of this heterophase interface. This last has been taken as a convenient average representative of interfaces in the phase-separated alloy in which phases at equilibrium are expectedly bounded by the following low-index interfaces sorted in ascending excess energy order, (111), (100) and (110). GCMC calculations at different temperatures included relaxations of atom positions, of the system volume and of the overall composition at fixed the difference between chemical potentials of the two species, *δμ*. The each time temperature dependent *δμ* has been set to the value driving a bulk AgCu solid solution to the solubility limit^42^. Upon convergence, interfacial and bulk regions reach the respective equilibrium compositions, revealing that the concentration of species at the interface is practically temperature independent, whereas bulk regions in this bi-crystalline model evolve toward the experimental solubility limits at each temperature. This finding shows that interfacial composition profiles are tightly related to the segregation of species, weakly changing on increasing the temperature, which justifies using as an input for the PF simulations the constant value, *κ* = 1.5 eV.nm^−1^ obtained as indicated above^33,42^. An additional approximation is made by maintaining the interfacial stiffness under irradiation at the same value, which is equivalent to admit that effects of thermal segregation of species at interfaces and species relocation under irradiation superimpose linearly. In the PF modeling, this value of *κ* has served for fixing the spatial correlation length of the coarse-grained compositional field, *ξ*, making this procedure the pivot connecting the scale of space of GCMC simulations (atomic) to that of PF simulations (mesoscopic). This is the ground of the multiscale modeling of phase separation phenomena investigated in the present work. In this context, it is worth noting that within the PF framework, $\xi \simeq \sqrt{\kappa /|{a}_{2}|}$, yielding, *ξ* ∼ 0.02 nm, a length comparable to the atomic spacing in AgCu much inferior to the correlation length of concentration fluctuations allowed by the Landau-Ginzburg phenomenology. Though singular, this result is the price to pay for fitting the composition values of the atomic scale modelling, in the bulk and at the interface, via the continuous and derivable functions solving the PF equations. Thereby, atomic modelling and PF lead to compositions in bulk phases identical to these obtained in the experiment. However, the quality of the fit does not attest for applicability of the PF modeling at the atom scale.

A final remark is about the deliberate choice made in this work, to consider that interfaces between phases are semi-coherent, which is justified given the mismatch between Ag and Cu lattices, but is certainly not true at the very beginning of the alloy decomposition and could not apply to states close to equilibrium, where incoherent relationships between phases may exist. However, the work focuses on stationary phases under irradiation (disrupted growth) that can be reasonably considered in a semi-coherent relationship whereas no experimental evidence has been found in the literature reporting on observations of incoherent precipitation in the range of temperatures and irradiation flux values used in the present study.

#### The irradiation term

Displacement cascades have been simulated within the Binary Collision Approximation (BCA) via the MARLOWE package^43^. Although displacement cascades thereby investigated do not account for atom vibrations and multiple collisions, such simulations have proven yielding on average a realistic description of atom relocation in the ranges of PKA energy and irradiation flux covered by ion mixing experiments in the ballistic regime. In particular, estimation of the effective cross-section of relocation events, *σ*^*r*^, and the ballistic displacement probability, *p*_*R*_, have proven consistent with MD simulations of copper irradiation in the range of energies of Primary Knock-on Atoms (PKA) accessible by this technique. The proof has also been given that the relocation range, *R*, is ion energy and flux independent, involving exclusively the chemical characters of incident ions and of the atom species present in the target. The detailed justification of the irradiation model used in this work can be found in^44^.

#### Equilibrium and irradiation mobilities

Under irradiation, the atomic mobility, *M*, has been evaluatedvia an hybrid numerical and analytical method embodying both the mobility terms, at thermal equilibrium and under irradiation where additional diffusion relates to the increase of point defect concentrations. At low and intermediate temperatures, the first term is predominantly vacancy-controlled and, since values of vacancy migration energies in Ag and Cu are close one to each other, has been conveniently approximated by the equilibrium mobility of vacancies in copper. For evaluating the second term, the average number of Frenkel pairs produced in displacement cascades has been obtained by using the MARLOWE package without recombinations. Since the time scales of point defect migration and of phase growth are very different, stationary irradiation defect concentrations have been estimated by using the chemical model of Sizmann, in the recombination regime, assuming that recombination is exclusively taking place at the interfaces between phases^45^. Elimination of defects at sinks consists in this model in a linear relation between recombination and the respective concentrations of point defects with the proportionality coefficient relating to the average size of precipitates^46^. The method would require downscaling of micro-structural data into the point defect model yet, due to the symmetry of Sizmann’s model, this coefficient does not appear in the stationary expression of defect concentration, which evaluation is thereby considerably simplified. Finally, it is worth noting that irradiation accelerates substantially mass transport below T = 900 K, whereas above this temperature thermal diffusion takes increasingly over^42^.

## Results

### Stationary state microstructures and patterning

Under irradiation, the forces driving the evolution of the alloy originate from ordering and disordering processes. Ordering proceeds by means of vacancy-mediated bulk diffusion and drives the alloy toward phase separation, because of the repulsive interactions between atom species. Disordering is caused by the ballistic displacements occurring within the collision cascades and tends homogenizing the alloy locally. It has been established that under the parallel action of the two processes non equilibrium stationary states can emerge when ordering and disordering come to balance one another. Example is given by the the dissolution of precipitates occurring under irradiation whenever the disordering rate overcomes that of thermal coarsening^24,25^. In the following, attention is given to patterning, that is the emergence of stationary, periodic distributions of precipitates in a decomposing alloy.

#### Stationary state criterion

**Figure 1:**
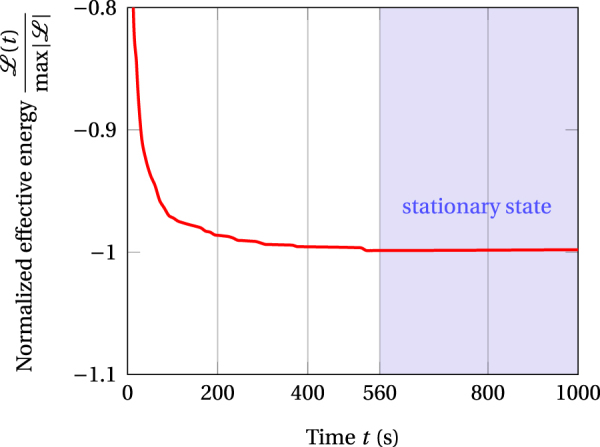
Typical time evolution of the reduced free energy, ${\mathcal L}$, toward the stationary state corresponding the minimum, ${\mathcal L} /{\rm{\min }}\,\{ {\mathcal L} \}\to -\,1$, reached in this case for elapsed time, *t* > 560 s (shaded domain).

In decomposing alloys, the thermal coarsening of precipitates is disrupted by io irradiation and leads to a variety of stationary microstructures that differ from these emerging under thermodynamical equilibrium conditions^25^. These out of equilibrium states correspond to the minima of the Lyapunov function, ${\mathcal L}$, embodying thermodynamical and ballistic effects (Equation 2, Fig. 1). This legitimates considering this function as an effective free energy of the system and the microstructure emerging upon convergence to a minimum as the signature of the corresponding stationary state ($t\simeq 560$ s in Fig. 1).

#### Flux and temperature as controlling parameters of stationary microstructures

**Figure 2:**
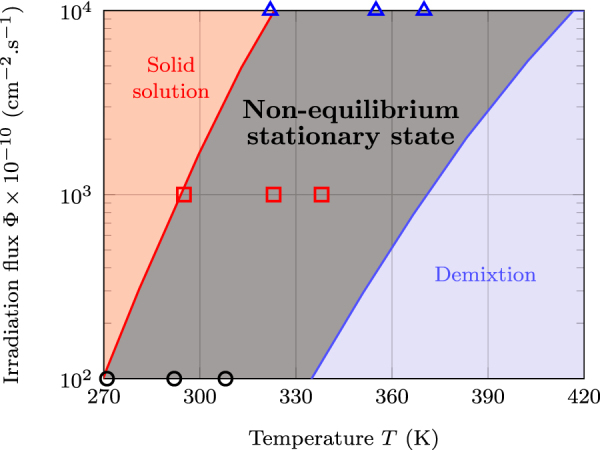
Pseudo-phase diagram for the silver-copper system irradiated with 1 MeV krypton ions displaying from left to the right the domains of the solid solution, of patterning and of demixtion^33^. In the patterning domain, flux values used in the present work correspond respectively to: Φ = 10^12^ cm^−2^ s^−1^ (open circles), Φ = 10^13^ cm^−2^ s^−1^ (open squares), Φ = 10^14^ cm^−2^ s^−1^ (open triangles).

**Figure 3:**
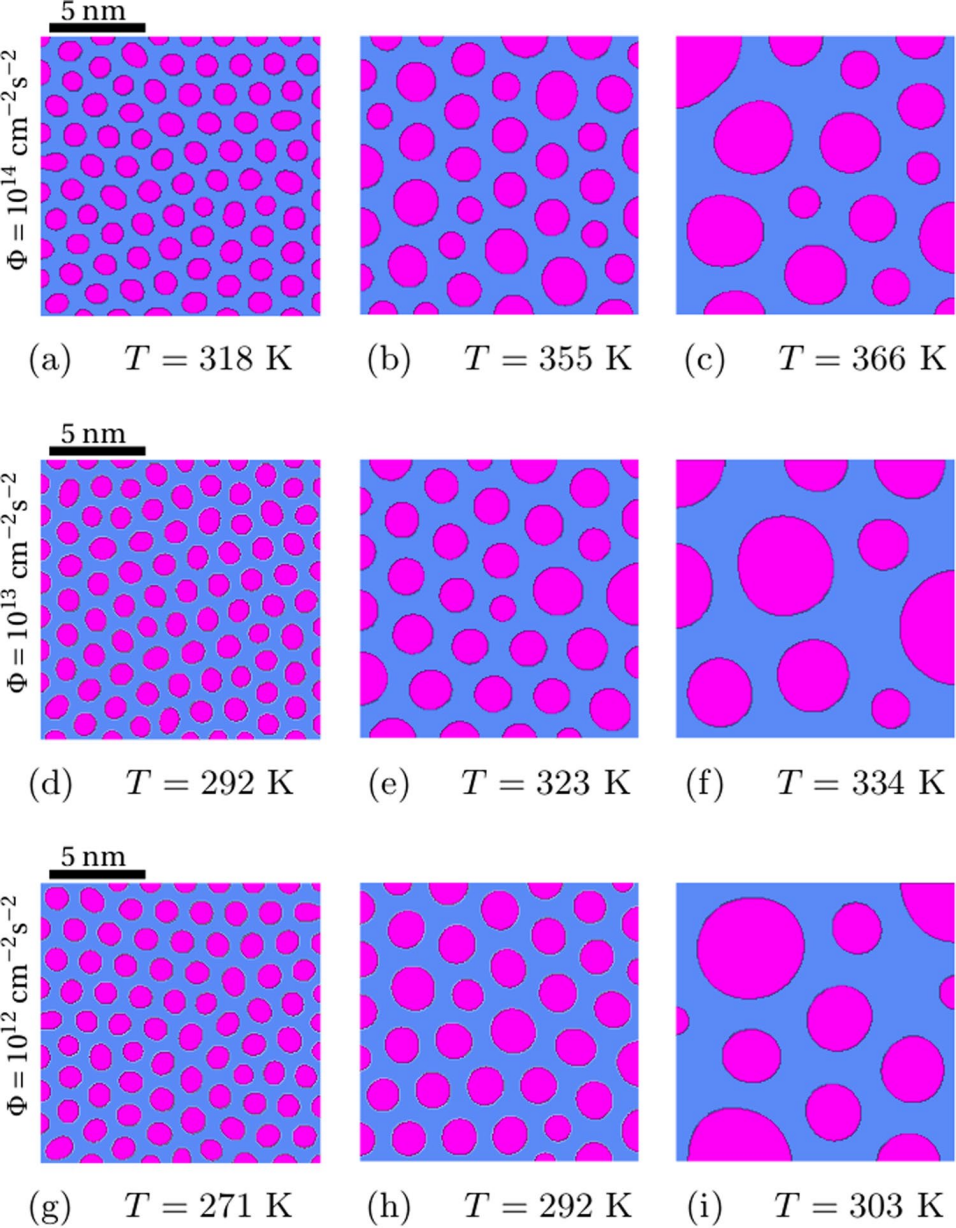
Irradiation microstructures in AgCu at different temperature and flux values (15 nm × 15 nm). Iso-concentration representation, disks: Ag precipitates, background: Cu matrix. Top: Φ = 10^14^ cm^−2^ s^−2^, middle: Φ = 10^13^ cm^−2^ s^−2^, bottom: Φ = 10^12^ cm^−2^ s^−2^ (1 MeV krypton ions).

Demange *et al*.^33^. have shown recently that the temperature and the irradiation flux are the parameters controlling exclusively the kind of stationary microstructures forming under ion irradiation. Thereby, a pseudo-phase diagram can be drawn in the temperature-flux plane revealing that all the possible stationary microstructures are organized within domains delimited by frontiers, which crossing would trigger micro-structural transitions (Figs 2, 3). This classification of irradiation microstructures is not new since it has been first proposed in a seminal work by Adda *et al*.^47^. Confirmation of the validity of this classification has been obtained by Barbu *et al*.^48^ who have explored the limits of radiation induced precipitation of *α* particles in solid solutions of NiSi with various compositions. Finally, Ye *et al*.^3^. have modeled the pseudo-phase diagram of alloys evolving under irradiation. Figure 2 shows that at low temperatures and high ion fluxes, where ballistic disordering predominates, the stationary state is a fully disordered solid solution, whereas high temperature and low ion flux values trigger phase separation since, in this case, ballistic effects do not counterbalance thermal diffusion. At intermediate flux and temperature values, coarsening is disrupted and a variety of microstructures are expected forming. In all these situations, the competition between irradiation and diffusion is the controlling factor as is shown by molecular statics studies where, in absence of thermal diffusion, the irradiation-induced mass transport triggers the formation of complex ordered structures. Previous diffraction studies by Wei *et al*.^26^ of the patterned microstructures forming in a concentrated AgCu alloy submitted to 1 MeV krypton ion irradiation reveal consistent with the above statements.

#### Stationary microstructures in the patterning domain

With the flux values given in Fig. 2 and temperatures lying within the patterning domain, ranging from 271 K to 366 K, the various stationary microstructures forming are displayed in Fig. 3. Disk-like phases are precipitates made principally of silver within a copper matrix represented by the background carrying a different contrast^24,25,33^. Moreover, this figure shows that at the lower temperatures (Fig. 3a,d,g), phase nanoparticles are practically mono-disperse in diameters, which provides a basis suitable for manufacturing materials with the desired mechanical response, provided the correspondence is established between the topology of the microstructure and the appropriate mechanical observable. On increasing the temperature, the dispersion of precipitate diameters increases as does the distance between them (Fig. 3 c,f,i). In total, this behavior is compatible with the theoretical and numerical investigations by Chen and Khachaturyan^49^ and by Abraham^50^.

### Microstructural design

#### Size of precipitates

**Figure 4:**
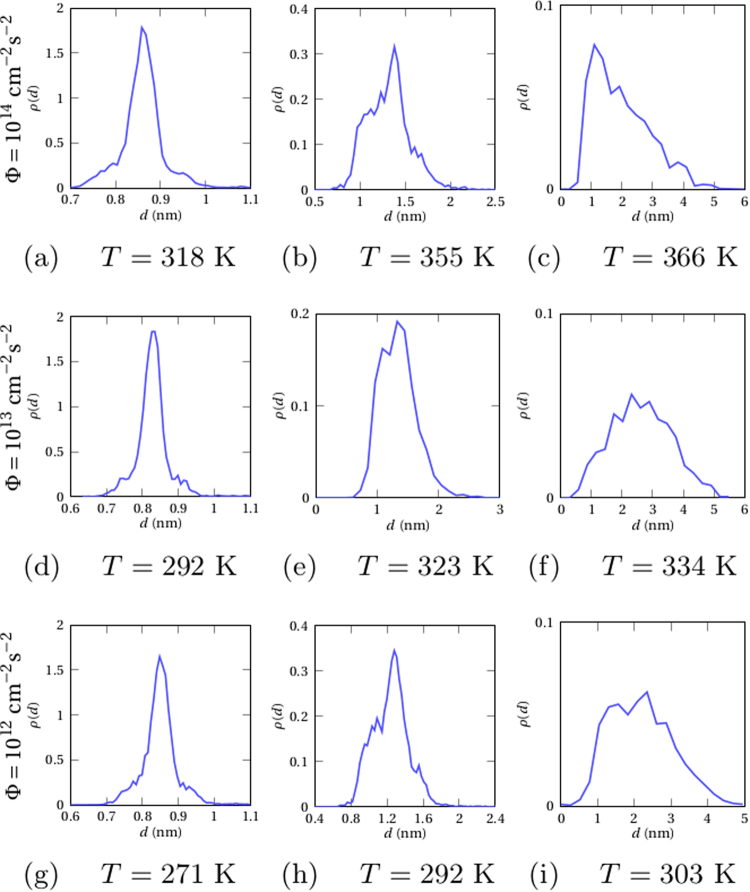
Diameter distribution of quasi-circular Ag precipitates produced under irradiation with 1 MeV krypton ions at different temperatures and flux values: Φ = 10^14^ cm^−2^ s^−2^ (top), Φ = 10^13^ cm^−2^ s^−2^ (middle), Φ = 10^12^ cm^−2^ s^−2^ (bottom).

The qualitative topological trends of stationary microstructures illustrated in Fig. 3 have also beenquantitatively studied as functions of the flux and of the temperature by computing the evolution of *n* = 200 initial random configurations of the solid solution at the critical composition, *c*_Ag_ = 0.39. Thereby, statistically meaningful averages of stationary particle diameters and composition fields have been obtained (Fig. 4). The former can be conveniently fitted by gaussians in confirmation of the statement made above that, at low temperatures, the size distributions of particles are mono-disperse. Such microstructures are fully characterized by specifying the average values of the composition and of the diameter of precipitates. At higher temperatures, by comparison to the case above, diameter distributions are enlarged, asymmetric, and heavily tailed, indicating that the relative proportion of large precipitates increases on increasing the temperature (Figs 3 and 4, right column). Considered all together, these results suggest that, under ion irradiation, composition modulations amplifying to form stationary microstructures extend over a pretty narrow range of wavelengths in difference with radiation-induced dissolution of precipitates during annealing, where coarsening is disrupted above a critical size value^51,52^. Thus, ion irradiation reveals well adapted to tailoring of quasi-mono-disperse distributions of precipitates as needed for microstructural design.

**Figure 5:**
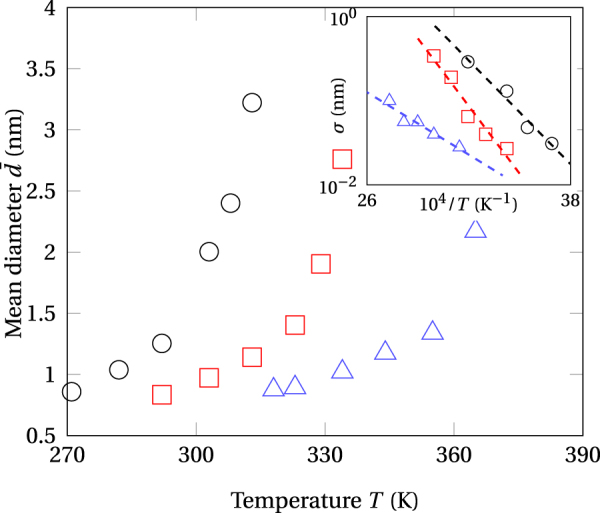
Average diameter of Ag precipitates, $\bar{d}$, as a function of the temperature and the irradiation flux. Open symbols: present work, Φ = 10^12^ cm^−2^ s^−2^ (circles), Φ = 10^13^ cm^−2^ s^−2^ (squares), Φ = 10^14^ cm^−2^ s^−2^ (triangles). Insert: Arrhenius plot of the standard deviation of Ag-precipitate diameters, *σ*.

In complement to Figs 4, 5 displays the mean diameter of stationary precipitates as a function of the temperature and the ion flux. It can be seen that the values are nanometric, amounting about twice the interfacial thickness at the frontier of phases (Cf. paragraph 2.2.2,^33^), which indicates that the predicted microstructures are closer to the expected stationary modulations of the composition in early stages of the spinodal decomposition^35^ than to an assembly of bulk phases. Interestingly, the conclusions of atom probe tomography observations in thermally aged stainless steels show that Cr precipitates as small as 0.8 nm form in the ferrite phase^53,54^, akin to these predicted by phase-field simulations of aging effects in a CuNi alloy^55^. KMC simulations from the literature provide qualitative support to the findings listed above and to the trends illustrated by Fig. 5 showing that the size of precipitates increases on increasing the temperature or by decreasing the irradiation flux^28^. Finally, the inset in this figure shows that the dispersion of precipitate size is thermally activated, a behavior tightly relating to the thermal mobility contribution underlying the phase separation.

#### Composition profiles, amplitudes and periodicity

**Figure 6:**
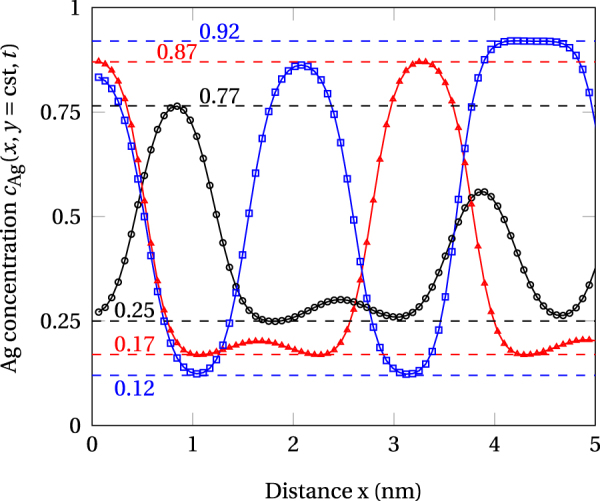
Ag concentration profiles as functions of the temperature at flux value, Φ = 10^13^ cm^−2^ s^−2^: *T* = 292 K (open circles), *T* = 303 K (full triangles), and *T* = 313 K (open squares).

The phase-field simulations performed in this work yield also informations about the composition and the periodicity of stationary precipitates forming under ion irradiation, which are expected closely relating to the mechanical behavior of the alloy^56^. Figure 6 displays the profiles of Ag concentration as a functions of the temperature at flux value, Φ = 10^13^ cm^−2^ s^−2^. It appears that the Ag content within precipitates exceeds by far the solubility limit at equilibrium of the bulk alloy^34^, whereas the concentration profiles adopt a nearly sinusoidal shape at low temperatures. At high temperatures, the figure indicates the formation of strongly segregated interfaces with Ag concentration close to that predicted for (100) interfaces (Cf. paragraph 2.2.2,^33^). This is in agreement with Monte-Carlo simulations, showing that under irradiation the temperature is the controlling parameter for the position dependent composition across an (100) interface, moving from segregated toward sinusoidal modulation profiles^8^. Moreover, on the experimental side, this behavior is reminiscent of the observations of intermixing produced during irradiation by energetic ions^57^ and matches the observations made by Atom Probe Tomography (APT) which showed the formation of quasi-periodical profiles of composition in Ag-40Cu after aging at 473 K^58^.

**Figure 7:**
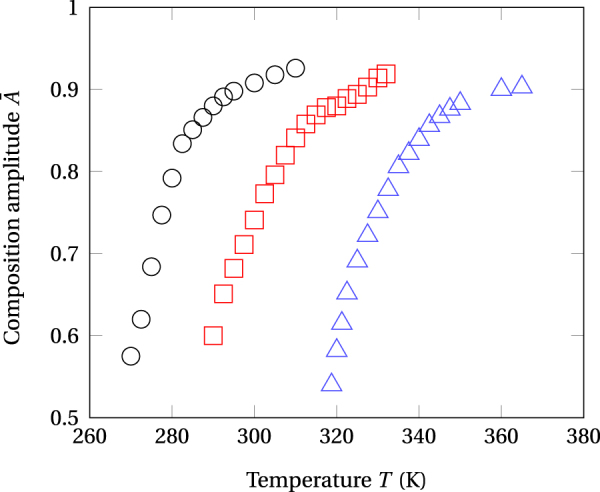
Amplitudes $\bar{A}$ of composition modulations as functions of the temperature and ion flux values. Open circles: Φ = 10^12^ cm^−2^ s^−2^. Open squares: Φ = 10^12^ cm^−2^ s^−2^. Open triangles: Φ = 10^13^ cm^−2^ s^−2^.

**Figure 8:**
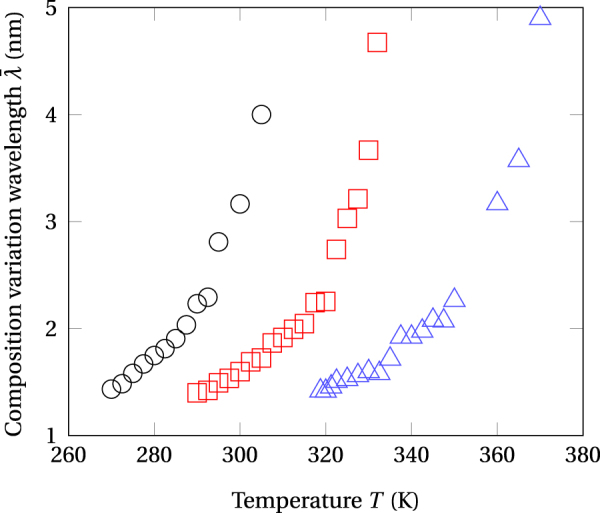
Dominant wavelengths $\bar{\lambda }$ of composition modulations as functions of the temperature and ion flux values. Open circles: Φ = 10^12^ cm^−2^ s^−2^. Open squares: Φ = 10^13^ cm^−2^ s^−2^. Open triangles: Φ = 10^14^ cm^−2^ s^−2^.

Computed composition amplitudes, $\bar{A}$, and modulation wavelengths, $\bar{\lambda }$, are respectively displayed as functions of the temperature and ion flux values in Figs 7 and 8. Amplitude maximum values reveal much larger than observed in after annealing studies of spinodal decomposition in AgCu, $\bar{A}=0.3$^58^. In addition, the wavelengths of stationary composition modulations investigated in the present work are much smaller the values obtained in studies of thermal aging in alloys^59–62^. It is therefore concluded that ion irradiation yields stationary precipitates with enhanced compositions much finely distributed than these produced by thermal annealing, adding thereby better tuning flexibility to microstructural design.

### From the irradiation induced microstructure to spinodal hardening

Precipitation in the spinodal decomposition domain changes the mechanical behavior of alloys through the interaction of mobile dislocations with precipitates impeding their motion. This “spinodal hardening” contributes differently to the material strength depending on the degree of advancement of the precipitation reaction: in the early stages of precipitation significant hardening is observed, which is followed by a saturation regime and, sometimes, a strength decrease with further evolving the microstructure^63–66^. Despite longstanding efforts, no widely accepted global modeling of this behavior has emerged as is attested by the numerous models proposed in the literature (Mott & Nabarro^67^, Cahn^56^, Ardell^68^, Friedel^69^, Ghista & Nix^70^ etc.). At least, Cahn’s theory of spinodal hardening convincingly describes the evolution of the mechanical property at the very first stages of precipitation. In this model, applied to cubic structures deforming via dislocation glide on (111)[110] slip systems, the Critical Resolved Shear Stress (CRSS), *τ*, is shown proportional to ${\bar{A}}^{2}\bar{\lambda }$, where $\bar{A}$ and $\bar{\lambda }$ represent the average values of the amplitude and wave length of composition fluctuations computed in Fig. 7. Upon increasing the size of precipitates, dislocations overcome the obstacles via the Orowan mechanism and the CRSS decay is predicted proportional to ${\bar{A}}^{\mathrm{1/3}}{\bar{\lambda }}^{-\mathrm{2/3}}$. Experimental confirmation of the model has been provided by Schwartz & Plewes^71^, who observed that the CRSS increase of a Cu-9Ni-6Sn alloy undergoing spinodal decomposition is strictly proportional to ${\bar{A}}^{2}\bar{\lambda }$, whereas the model does not reveal capable of satisfactorily describing the late stages of the microstructure evolution. Similar findings have been reported on Cu-5at%Ti by Miyazaki *et al*.^72^ and by others for various systems^73,74^. It therefore appears that Cahn’s theory applies strictly to the periodic microstructures emerging at the beginning of spinodal decomposition, not to their long term evolution. The applicability of the theory to the microstructures modeled in the present work is further commented on in the following section.

**Figure 9:**
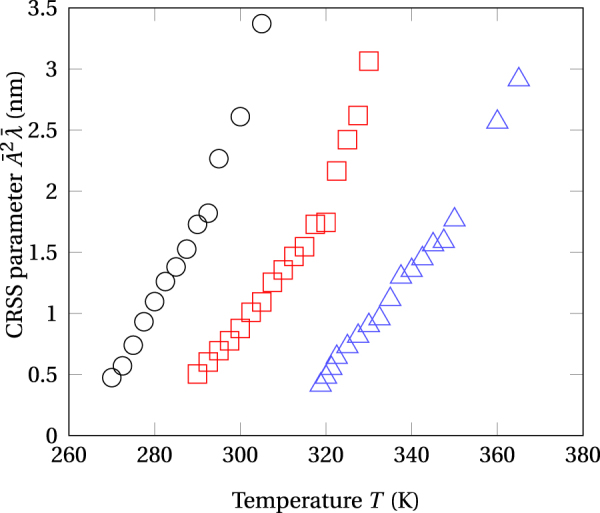
CRSS parameter, ${\bar{A}}^{2}\bar{\lambda }$, as a function of the temperature for three values of the irradiation flux with 1 MeV krypton ions. Open circles: Φ = 10^12^ cm^−2^ s^−2^. Open squares: Φ = 10^13^ cm^−2^ s^−2^. Open triangles: Φ = 10^14^ cm^−2^ s^−2^.

A main finding of the present study is that composition variations in the decomposing alloy are nearly sinusoidal in the patterning regime (low temperatures), which strongly suggests that Cahn’s theory can be used for evaluating the CRSS associated to such patterned microstructures. Accordingly, the expression of the CRSS *τ* is given by:

3 $$\tau =(\frac{2\pi {\eta }^{2}{Y}^{2}b}{C\gamma }){\bar{A}}^{2}\bar{\lambda }=\alpha {\bar{A}}^{2}\bar{\lambda }\mathrm{.}$$

where, ${\bar{A}}^{2}\bar{\lambda }$ is hereafter referred as the CRSS parameter, $\eta =\partial \,\mathrm{ln}\,a/\partial c$ is the distortion parameter, *a*, represents the lattice parameter at a given concentration, *c*, *Y*, is the Young’s modulus, *b*, the Burgers vector, *γ*, the line tension of glide dislocations and *C*, a coefficient that equals, $3\sqrt{6}$ or $\sqrt{2}$, for screw and edge dislocations respectively. Figure 9 shows the evolution of ${\bar{A}}^{2}\bar{\lambda }$ from the simulations, as a function of temperature for different irradiation fluxes. In qualitative agreement with experiments, the graphs representing the evolution of *τ* with the temperature are shifted toward the higher temperatures on increasing the irradiation flux. However, it must be emphasized here on that only the contribution of the spinodal decomposition to the strength has been considered and that other causes may further harden the alloy as would expectedly happen when the dislocation density increases in response to a flux increase. This adds to the possible failure at high temperatures of the CRSS prediction via equation 3 since the concentration profile is not sinusoidal in shape anymore as is shown in the present work. Nonetheless, the above results constitute a first attempt relating PF predicted mesoscopic microstructures to the mechanical property. Further effort is required for satisfactorily modeling the evolution of the CRSS under irradiation and for clarifying the origins of hardening regimes observed in irradiated alloys.

## Conclusive Remarks

In section B.4, it is mentioned that the present modeling assumes that interfaces between emerging phases are the sinks where point defects can recombine. For the sake of simplicity, other recombination mechanisms involving cavities and dislocation loops are neglected. Ongoing work is devoted to extending the present model by including in the free-energy functional describing the evolution of the alloy under irradiation the ingredients capable to trigger the formation of these micro-structural features. The present work should be considered as a first attempt to rely irradiation microstructures with the associated mechanical behavior of the alloy. In this context, it is worth examining the reasons for applicability of Cahn’s theory in the light of the finding that irradiation leads to the disruption of coarsening of phase particles. Controlling the temperature, the irradiation flux and the overall composition of the initial solid solution allows for fixing the stationary values of the diameters and the composition of the phase particles. Thereby, the micro-structural state can be kept close to that targeted in the theory of spinodal hardening, that is to say close to the microstructure at the very beginning of the phase separation. How far this theory is able to predict the mechanical resistance of the alloy, upon increasing the values of the composition and the diameters of phase particles, is an open question that only experiments alike these realized by Schwartz & Plewes^71^ and Miyazaki *et al*.^72^ are capable to answer, with clear benefit for further accurate modeling of the mechanical property of irradiation microstructures.

In conclusion, the present work is a first attempt to predict the mechanical behavior of irradiation microstructures, modeled by using the multiscale phase field approach developed previously^33^, and to establish a connection with the experimental conditions, temperature and irradiation flux. Among the two types of stationary irradiation microstructures found in the decomposing AgCu alloy, periodic composition modulations forming closely packed diffuse particles of nanometric size at low temperatures and the disordered collection of larger precipitates with strongly segregated interfaces at high temperatures, a credible prediction of the mechanical response is only possible for the former by using the Cahn’s model of spinodal hardening^56^. The mechanical behaviour of the latter microstructures, is still an open question. Forthcoming work will focus on short-range order effects in such microstructures, possibly controlling the mechanical response of the alloy^50,75^.
